# Effect of ionizing radiation on the shear bond strength of two different adhesive systems in primary teeth. in-vitro study

**DOI:** 10.1186/s12903-024-04996-y

**Published:** 2024-10-21

**Authors:** Nourhan Mahmoud Shahin, Basma Mahmoud Nagi, Amin El Sayed Amin, Amira Saad Badran

**Affiliations:** 1 Department of Pediatric Dentistry and Dental Public Health, Faculty of Dentistry, Ain Shams University, African Unity St, Cairo, 11566 Egypt; 2 Department of Radiation Oncology and Nuclear Medicine, Faculty of Medicine, Ain Shams University, Cairo, Egypt

**Keywords:** Radiotherapy, Ionizing radiation, Dental adhesives, Primary teeth, Shear bond strength

## Abstract

**Background:**

Radiotherapy is a treatment modality used for head and neck cancer patients. It has a negative influence on bonding strength of adhesives. Little information is available about the effect of radiotherapy on bonding strength of adhesives in primary teeth. Therefore, this in vitro study aimed to detect the best adhesive system and ideal time to apply restorations in primary irradiated teeth regarding adhesive shear bond strength.

**Methods:**

Dentin samples from primary teeth were randomly assigned to four groups based on restoration application time and radiation exposure, (G1: control, G2: preradiation, G3: 24 h postradiation, and G4: 6 months postradiation) with 20 samples per group. These groups were further divided into 2 subgroups according to the adhesive system used, with 10 samples per subgroup. (1) 3M™ Single Bond Universal Adhesive (SB), (2) 3M AdperSingle Bond 2 (AS). Samples were exposed to gamma radiation from a cobalt-60 machine. One shot of 60 Gy of radiation was delivered. Then samples were subjected to a shear bond strength test. The load was applied until failure and the maximum load was recorded. Numerical data are presented as mean and standard deviation values, then distributed according to Shapiro–Wilk test or Levene's test and analyzed via two-way ANOVA. The significance level was set at *p* < 0.05 for all tests. Statistical analysis was performed with R statistical analysis software version 4.4.1 for Windows (Team RC, R: A language and environment for statistical computing. R foundation for statistical computing, 2023).

**Results:**

Compared with the AS subgroup, the SB subgroup presented significantly greater values (*p* < 0.001). For SB, there was no significant difference among the G1 and G4 groups (*p* > 0.001). However, there was a significant difference between G1, G2, and G3 (*p* < 0.001, *P* = 0.025, *P* = 0.265 ns respectively), and G3 presented the lowest values. For AS, there was no significant difference between groups G1 and G4 (*p* =  < 0.001). Compared with the other groups, G3 presented a significant difference (*p* = 0.265 ns) and the lowest results.

**Conclusion:**

Restorations performed 24 h after radiation had lower bond strength than those performed six months after radiation. Regardless of the adhesive system used, SB performed better than AS in terms of the shear bond strength.

**Supplementary Information:**

The online version contains supplementary material available at 10.1186/s12903-024-04996-y.

## Background

Cancer is a critical health issue worldwide. According to global cancer statistics (2024), 20 million new cases were diagnosed in 2022, and 9.7 million deaths occurred in the same year; this number is predicted to reach 50 million by 2050 [1]. Head and neck cancer ranks seventh in the world, with approximately 660,000 new cases annually [2]. Thyroid cancers have been reported to be more common than head and neck malignancies in children [3].

The treatment can be very efficient, with a high chance of a cure. Surgical treatments are frequently used in cancer treatment, with other therapies including radiotherapy and chemotherapy [4].

Although radiotherapy has a high success rate, it has many side effects such as mucositis, xerostomia, soft tissue necrosis, osteoradionecrosis, trismus, progressive loss of the periodontal ligament, and radiation-related caries [5].

Before radiotherapy (RT), it is crucial to eradicate carious lesions and decrease the number of oral cavity infection foci. However, after RT for head and neck cancer (HNC), the risk of caries related to radiation increases. Hence, it is necessary to decrease the risk factors for caries [6].

Currently, composite resin is considered the most popular restorative material because it is aesthetically pleasing, has a highly polished surface, and has increased wear resistance compared with glass ionomers [7]. However, the clinical performance of this material under the effect of radiation and its overall success is a matter that needs further investigation.

Radiotherapy and its side effects which are related to the oral cavity can affect the bond strength of adhesives [8]. These effects are dependent on the mineral and organic components of the dental hard tissue [9].

The composition and morphology of primary teeth are diverse from those of permanent teeth; they are thinner and have a greater numerical density of rods, [10] abundant micropores, exposed prisms, major carbonate incorporation [11], and lower concentrations of calcium and phosphorus than permanent teeth [12]. These differences may result in different effects of ionizing radiation on the dental hard tissue of primary teeth.

In permanent teeth, the impact of ionizing radiation on the bond strength of etch and rinse and self-etch adhesives has been evaluated in numerous studies [13–19]. Some studies reported a decrease in adhesive strength to enamel and/or dentin, especially when restorations were applied after exposure to radiation [13, 14, 16, 17].

In 2020, Arid et al. [19] reported a decrease in the bond strength to dentin when irradiation was applied after restoration. However, other studies reported no significant reduction in the bond strength after exposure to radiation [15, 18].

Regarding the type of adhesive systems used, the literature reveals controversial findings. Some studies have reported that self-etch adhesives have higher values than etch and rinse adhesives when the bond strength of teeth after radiation is evaluated [15, 19, 20]. Other studies have reported that etch and rinse adhesive was better than self-etch adhesive [21, 22].

Additionally, Oglakci et al. in 2022 evaluated the shear bond strength of a universal adhesive and reported that the etch&rinse mode resulted greater bond strength to enamel than the self-etch mode when teeth subjected to radiation were used [23].

Recently, the market has developed numerous types of dental adhesives with different chemical compositions and different bonding strategies; unfortunately, little information is known about the effects of ionizing radiation on dental composite-adhesive systems in primary teeth.

Therefore, the purpose of this in-vitro study was to detect the most proper adhesive technique (a comparison between 3 M Adper single bond 2 adhesive [etch & rinse] and 3 M single bond universal adhesive [self-etch]) and the appropriate time to perform the adhesive restoration in the primary teeth of HNC patients in terms of adhesive shear bond strength.

The null hypothesis states that (1) Ionizing radiation does not affect the dentin bond strength of the adhesive systems when used on the dentin of primary teeth. (2) There is no significant difference between self-etch adhesive and etch and rinse adhesives when used on irradiated teeth.

## Methods

A power analysis was designed to ensure adequate power to apply a statistical test of the null hypothesis that there is no difference between different tested groups regarding shear bond strength. Cohen's f was chosen as the effect size for the calculation as it is appropriate for use in the analysis of variance (ANOVA) and is a standard metric for evaluating the magnitude of differences between group means [24]. It was calculated using the reported shear bond strength values for dentine adhesion in Table 2 of the referenced paper [19].

(From the reported values the pooled standard deviation was calculated using the following formula and was found to be (6.15).

$$\text{sd}\_\text{pooled}=\text{sqrt}\left(\left(\sum\left(\mathrm{n}\_\mathrm{i}-1\right)\ast\upsigma\_\mathrm{i}^\wedge2\right)/\left(\sum \mathrm{n}\_\mathrm{i}-\mathrm{g}\right)\right)$$

where: sd_pooled: Pooled standard deviation, n_i: Sample size of group I, σ_i: Standard deviation of group I, g: Number of groups.

The grand mean was calculated using the following formula and was found to be (15.86):

$$\upmu \_\text{G }=\Sigma (\text{w}\_\text{i }*\upmu \_\text{i})$$

where: μ_G: Grand mean, w_i: Weight for group I, μ_i: Mean of group i.

where the weights of all groups were (0.125).

The between-groups standard deviation was calculated using the following formula and was found to be (6.00):

$$\text{sd}\_\text{between }=\text{ sqrt }(\Sigma (\text{w}\_\text{i }* (\upmu \_\text{i }-\upmu \_\text{G})^2))$$

where: sd_between: Between-group standard deviation, w_i: Weight for group I, μ_i: Mean of group I, μ_G: Grand mean.

Finally, Cohen's f was calculated using the following formula and was found to be (0.975):

$$\mathrm{f}=\text{sd}\_\text{between} / \text{sd}\_\text{pooled.)}$$

The sample size was calculated using the acquired effect size and by setting alpha (α) and beta (β) levels to (0.05), i.e., power = 95%, and was found to be [25] samples and it was a minimum estimation of the required sample size. To account for possible variability and failure during testing, the sample size was increased to 80 samples (i.e., 20 samples per group, with 10 samples per subgroup). Sample size calculation was performed using R statistical analysis software version 4.4.1 for Windows [26].

### Ethical aspects and samples

This study was exempt from ethical review by the Ain Shams University Research Ethics Committee (FDASU-REC), [ethics committee approval: FDASU-Rec EM112218] as it was an in vitro study that used teeth collected from anonymous patients who consented to the use of their extracted teeth for research purposes, and it’s a routine process as they are treated in an educational hospital.

Forty sound exfoliated human primary maxillary and mandibular molars or those extracted for orthodontic purposes were obtained from the Pediatric Dentistry and Dental Public Health Clinic, Faculty of Dentistry, Ain Shams University, and stored in distilled water at room temperature.

### Sample preparation

All teeth were debrided with a hand-scaler and polishing paste with a rubber cup mounted on a low-speed contra-angle handpiece. The teeth were then stored in distilled water at room temperature (23 ± 1°C) in an incubator [27].

Each tooth was sectioned mesiodistally into two halves under copious air‒water coolant spray by a diamond disk to produce 2 samples. The roots were cut off at the cervical line. The dentin surfaces were also wet abraded until the flat dentin surfaces were reached for use as a substrate for testing bond strength.

**Fig. 1:**
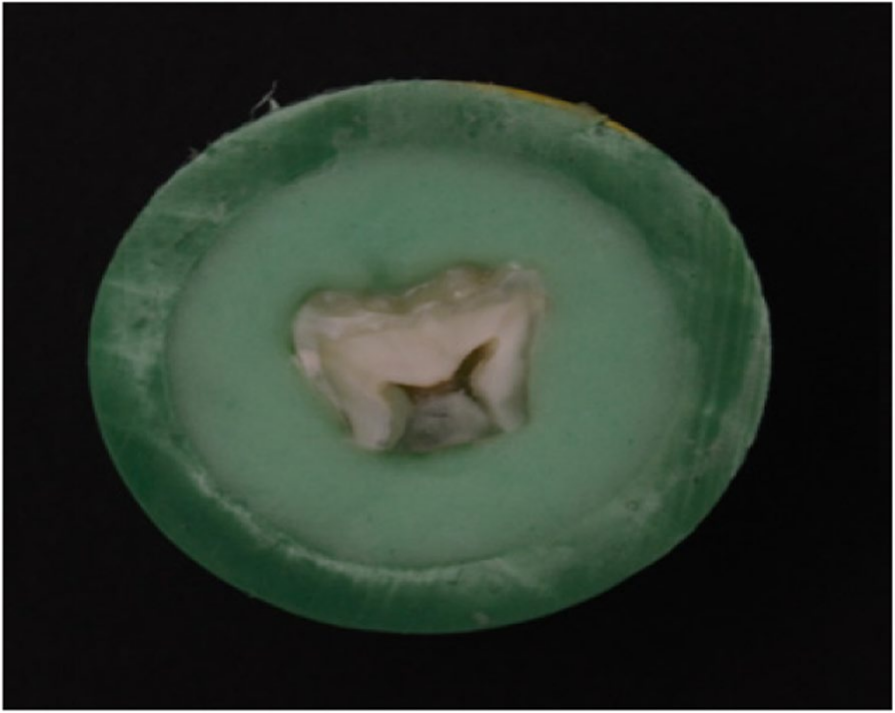
Specimen embedded in self-cured acrylic resin within polyvinyl ring

Polyvinyl chloride (PVC) rings with an internal diameter of 1.5 cm and height of 1 cm were fixed over a glass slab via a double-faced adhesive. The prepared sample was placed in the center of the ring with the labial enamel/dentin surface facing downward. A mixed cold cured acrylic resin (Acrostone, Egypt) was poured into the ring until it was flush with the upper edge of the tube. After the acrylic resin was set, each dentin sample was wet ground over #180 SiC paper to remove the excess acrylic resin material, if present. (Fig. 1) The prepared samples were subsequently kept in distilled water.

### Group assignment

**Fig. 2:**
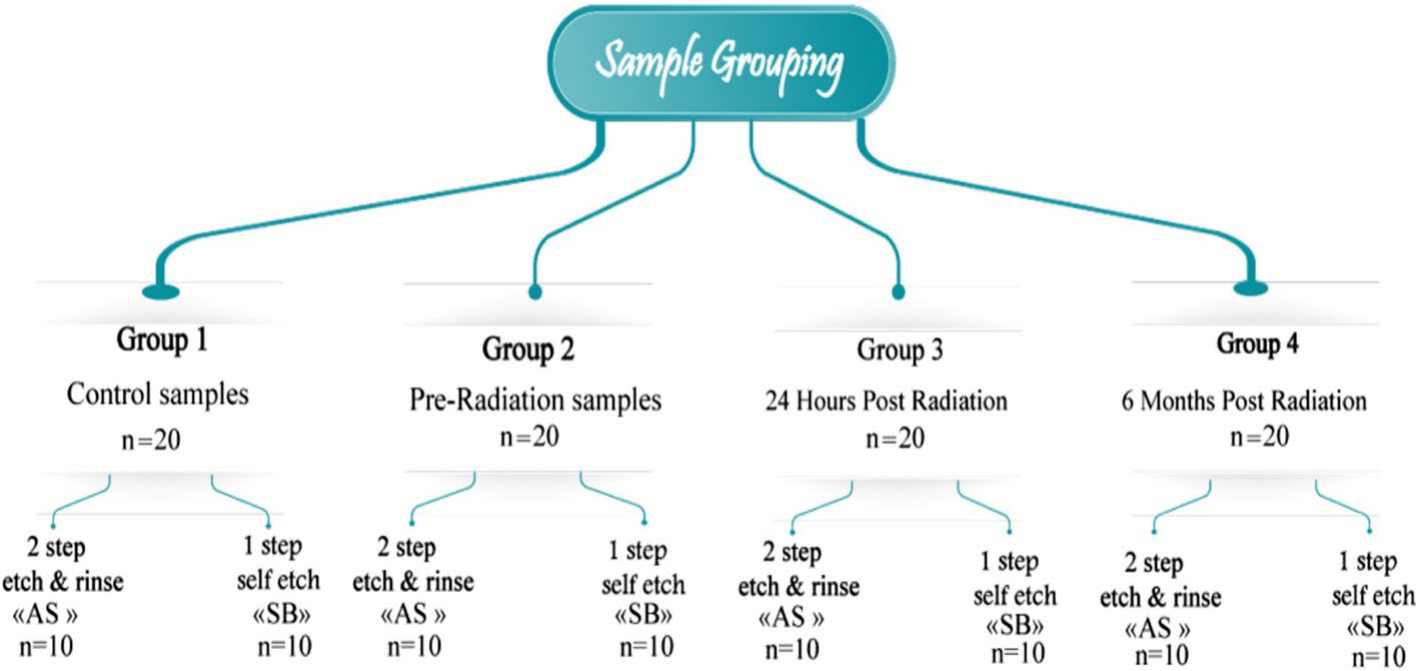
Flowchart of shear bond strength test grouping

A total of 80 samples were randomly assigned to the four main groups based on restoration application time and radiation exposure, (G1: control, G2: pre radiation, G3: 24 h post radiation, and G4: 6 months post radiation) with 20 samples per group. These groups were further divided into 2 subgroups according to the adhesive system used, with 10 samples per subgroup. (1) 3M™ Single Bond Universal Adhesive “Self-etch” (SB: 3M/ESPE, St. Paul, Minn., USA). (2) AdperSingle Bond 2 “Etch&rinse” (AS: 3M/ESPE, St. Paul, Minn., USA). (Fig. 2).

**Fig. 3:**
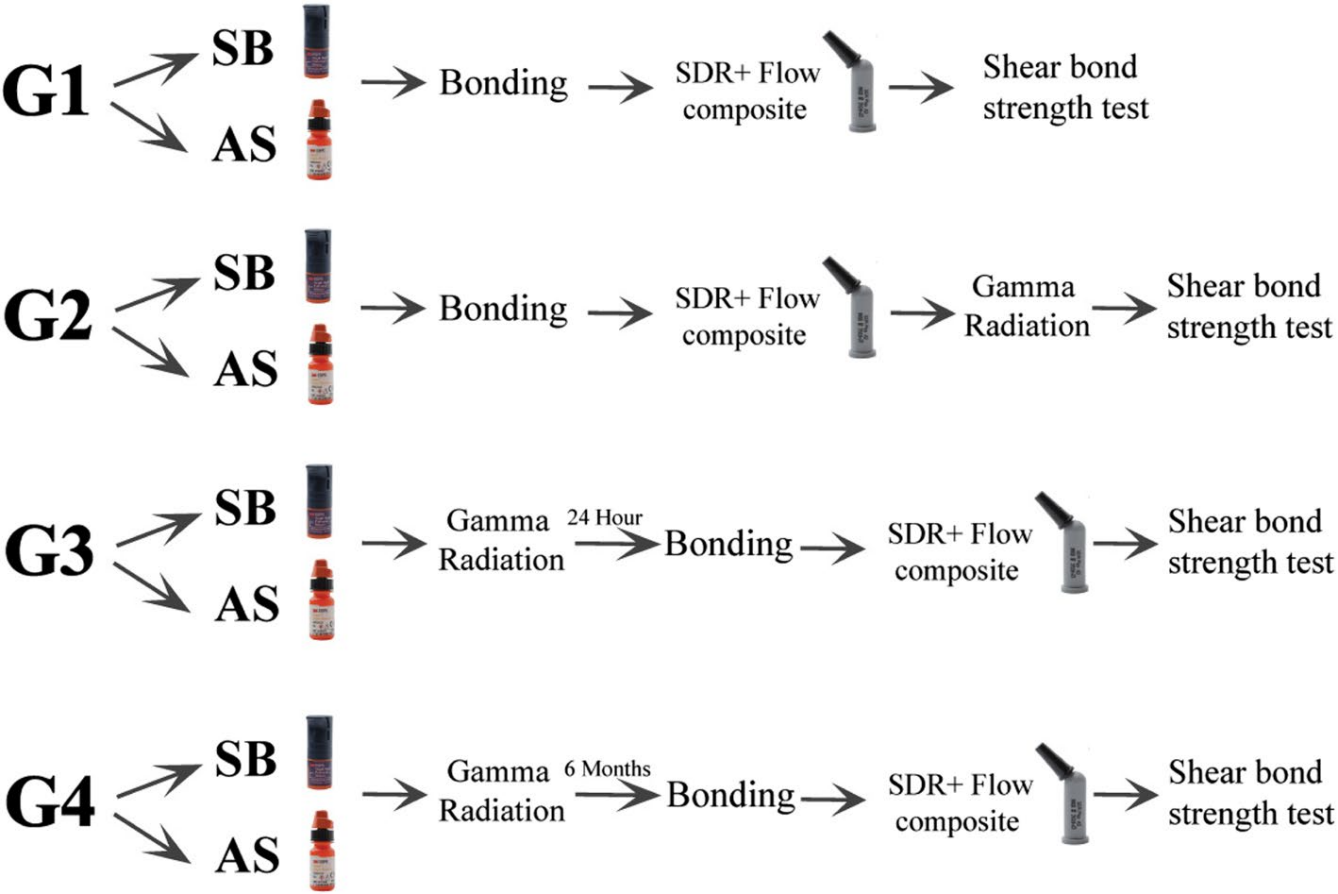
The flowchart of the experimental setup

The flowchart of the experimental setup is shown in Fig. 3.

### Restorative procedure

Adhesive systems were applied to the ground surfaces, according to the instructions of the manufacturer and the sample subgroups.

#### For subgroups (SB1, SB2, SB3, SB4)

**Fig. 4:**
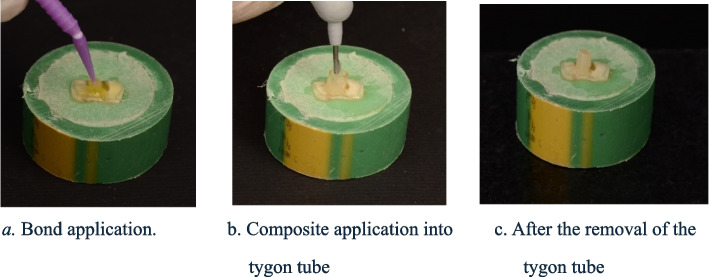
Restorative procedures and application of the adhesive system

3M Single Bond Universal Adhesive (3M, USA) was used, one layer of the bond was applied on the sample and rubbed for 20 s then air dried gently for approximately 5 s to evaporate the solvent. Then the tygon tube was placed over the uncured bond and the bond was cured for 10 s using the LED curing unit with an output of 1200 mW/cm2 “LED 3M ESPE Elipar TM”. SDR flow + composite **“**Dentsply Sirona, USA” was applied in one increment and cured for 40 s. Then the rubber Tygon tube was removed with a lancet. (Fig. 4).

#### For subgroups (AS1, AS2, AS3, AS4)

3M Adper Single Bond 2 adhesive (3M, USA) was used, Meta Etchant “Meta Biomed Europe GmbH, Wiesenstr, Germany” was applied for 15 s, rinsed for 10 s and blot excess water was removed by a cotton pellet as the surface should appear glistening without pooling of water.

The 2–3 layers of bond were applied to the etched dentin for 15 s with gentle agitation using a fully saturated applicator and gently air dryied for five seconds to evaporate the solvents. Then the tygon tube was positioned over the uncured bond and the bond was cured for 10 s using the LED curing unit with an output of 1200 mW/cm2 “LED 3M ESPE Elipar TM”.The SDR flow + composite **“**Dentsply Sirona, USA “ was applied in one increment and cured for 40 s. Then the rubber tygon tube was removed with a lancet. To ensure that the laboratory phases were uniform, the samples were coded.

### Irradiation process

**Fig. 5:**
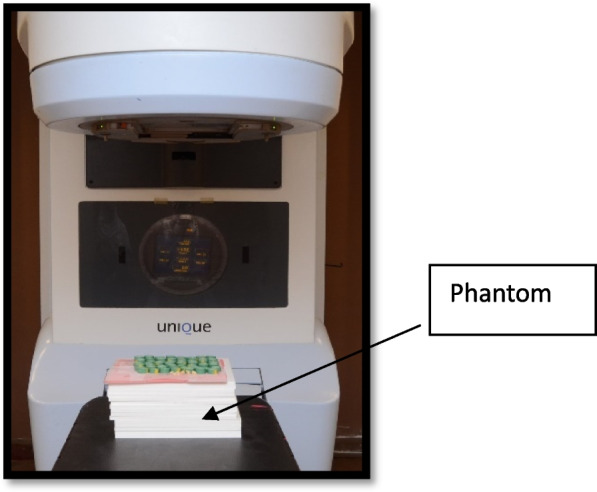
Samples placed over the phantom

**Fig. 6:**
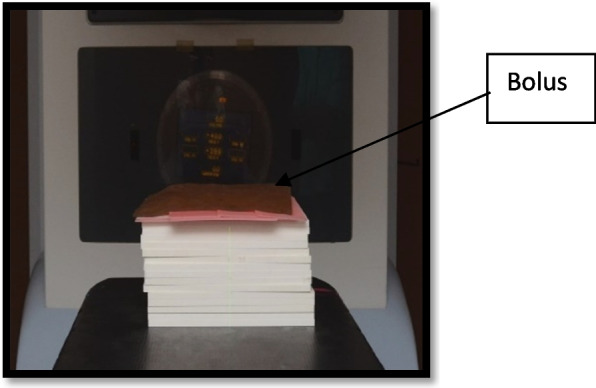
After the application of the bolus over the samples

To achieve complete side and back radiation scattering, the samples were exposed to radiation on a phantom with a height of 15 cm. During irradiation, the samples were covered with a bolus to build up the radiation dose. A cobalt-60 machine (Theraton Phoenix 60 cobalt Radiotherapy Treatment Unit – Theratronics International, Ltd., Atomic Energy of Canada, Ltd., AECL Medic al, Ontario, Canada) was used to emit gamma rays during the radiation process. The distance from the source head of the machine to the samples was about 80 cm. One dose of radiation totaling 60 Gy “Gray” was adjected and administered by the physician and it took about 15–20 min [28, 29] (Figs. 5 and 6).

For the pre-irradiation group (G2): The samples were irradiated after composite restoration application. Then the samples were kept in distilled water at 37°C in an incubator, after which shear bond strength testing was measured.

For the post-irradiation group (G3): The samples were irradiated and then kept in distilled water at 37°C in an incubator for 24 h. Then composite restoration was applied, and the shear bond strength was measured.

For the post-radiation group (G4): The samples were irradiated then kept in distilled water at 37°C in an incubator for 6 months, the water was changed weekly, the resin composite was applied, and shear bond strength testing was measured.

### Shear bond strength testing

**Fig. 7:**
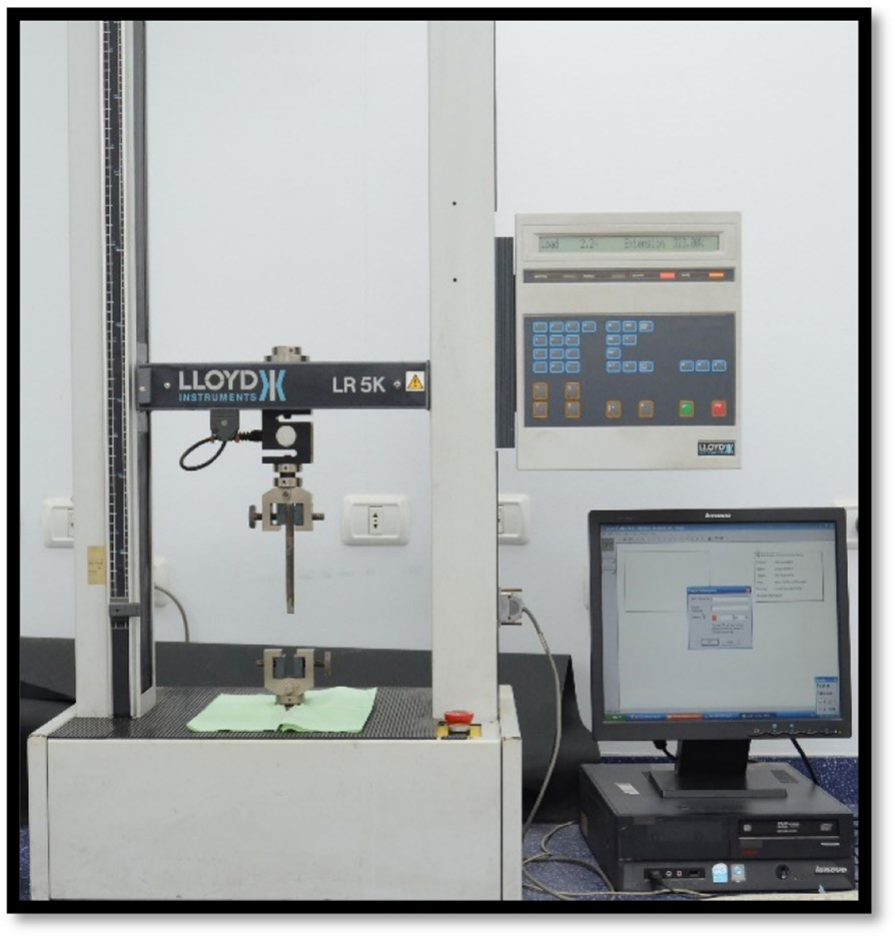
Universal testing machine

**Fig. 8:**
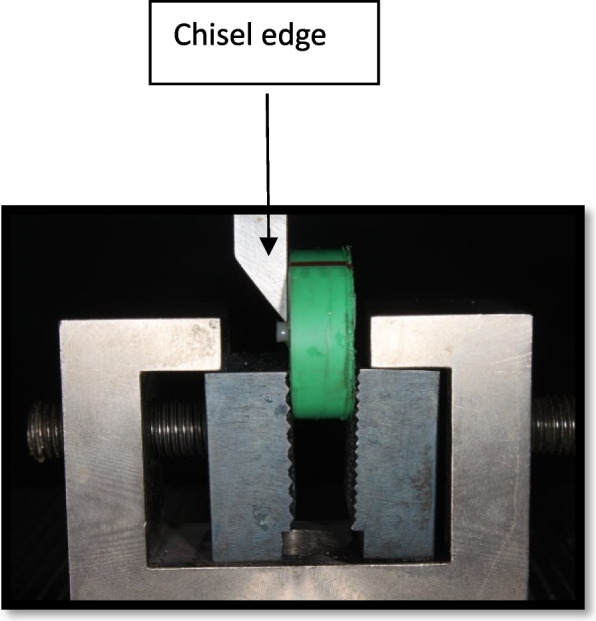
Sample setting in the universal testing machine

A specially constructed load applicator with a chisel edge was attached to the upper jig of a universal testing machine (LR5K series, LLOYD Instruments, Ltd., UK). Each sample was attached to the lower jig of the universal testing machine. The load was applied at a crosshead speed of 0.5 mm/min until failure, and the maximum failure load was recorded in Newtons and then converted to Megapascals. These values were subsequently used for statistical analysis. (Figs. 7 and 8).

### Statistical analysis

The data were normally distributed according to the Shapiro–Wilk test or Levene's test and were analyzed via two-way ANOVA. The significance level was set at *p* < 0.05 for all tests. Statistical analysis was performed with R statistical analysis software version 4.4.1 for Windows [26].

## Results

### Shear bond strength test

Two-way ANOVA revealed that irradiation timing and the adhesive system had a statistically significant effect on shear bond strength (*p* < 0.001) (Table 1),

**Table 1:** Comparisons and summary statistics of shear bond strength (MPa) for different irradiation conditions and adhesion protocols

Adhesion protocol	Shear bond strength (MPa) (Mean ± SD)				*P*- value
	**Control group** **Group “1”**	**Preradiation group** **Group “2”**	**24 h** **Post radiation group** **Group “3”**	**6 months post-radiation group** **Group “4”**	
**Adhesive 1 “SB”**	21.09 ± 3.74^A^	10.60 ± 2.41^B^	4.91 ± 1.61^C^	22.32 ± 3.63^A^	< 0.001*
**Adhesive 2 “AS”**	9.64 ± 2.38^A^	6.99 ± 1.13^A^	3.14 ± 1.11^B^	8.86 ± 2.55^A^	< 0.001*
***P* value**	< 0.001*	0.025*	**0.265 ns**	< 0.001*	

^*^Values with different superscript letters within the same horizontal row are significantly different *; significant (*p* < 0.05) *ns* nonsignificant (*p* > 0.05)

Group with "A" symbol is followed by group with "B" symbol then the lowest is group with "C" symbol

For adhesive 1 “SB”, there was a significant difference between the different groups (*p* < 0.001). The highest value was measured in group “4” (22.32 ± 3.63) (MPa), followed by group “1” (21.09 ± 3.74) (MPa) and then group “2” (10.60 ± 2.41) (MPa), whereas the lowest value was measured in group “3” (4.91 ± 1.61) (MPa). Post hoc pairwise comparisons revealed that samples in groups “1” and “4” did not significantly differ from each other but had significantly greater bond strengths than the other groups did (*p* < 0.001). In addition, the samples in group “2” had significantly greater bond strengths than did the samples in group “3” (*p* < 0.001). (Table 1).

For adhesive 2 “AS”, there was a significant difference between the different groups (*p* < 0.001). The highest value was measured in group “1” (9.64 ± 2.38) (MPa), followed by group “4” (8.86 ± 2.55) (MPa) and then group “2” (6.99 ± 1.13) (MPa), whereas the lowest value was in group “3” (3.14 ± 1.11) (MPa). Post hoc pairwise comparisons revealed that the bond strength of group “3” was significantly lower than those of the other groups (*p* < 0.001). (Table 1).

The interactions between the irradiation conditions and the adhesion protocol are presented in (Table 2).

**Table 2:** Effects of different variables and their interactions on the shear bond strength (MPa)

Variable	Sum of squares (II)	df	Mean square	*f*-value	*p*-value
Irradiation	1872.03	3	624.01	49.92	< 0.001*
Adhesion protocol	1147.93	1	1147.93	91.84	< 0.001*
Irradiation * Adhesion protocol	495.56	3	165.19	13.22	< 0.001*

^*^df degree of freedom, * significant (*p* < 0.05)

## Discussion

Cancer is a public health problem; its recognition has had a disastrous impact on human health and well-being has developed over the years [30]. RT is the most common treatment approach used for malignant tumors in the head and neck regions [31]. Nevertheless, it has adverse effects that are both short- and long-term. Infection, dysphagia, taste loss, and mucositis are short-term adverse effects. Trismus, radiation caries, and osteoradionecrosis are long-term adverse effects [32].

Therefore, a focused "multidisciplinary team approach" is the most potent way to manage HNC patients before, during, and following radiotherapy [25].

Despite the increasing prevalence of head and neck cancer patients in need of dental restorative treatment, clear internationally accepted guidelines are yet to be set for dental patients before and during their restorative treatment [18, 33]. Similarly, the optimum timing of treatment besides the effects of radiation on the tooth structure and bonding potential are still unclear [17].

Few studies have investigated the effects of radiation on the bonding strength of adhesives in primary teeth. Therefore, it is worth studying the effect of radiotherapy on the bonding strength of different resin composite adhesive systems in primary teeth when bonding after irradiation (postradiation) and before irradiation exposure (preradiation).

To provoke the effects of irradiation, a cobalt -60 machine was used as a source of X-rays, and the samples were irradiated with 60 Gy, which is common for HNC patients and is acknowledged as the critical dose beyond which changes occur [34, 35].

We did not use a cumulative fractionated dose protocol, as in standard head and neck cancer therapy (2 Gy per day, 5 days a week), [36, 37] as the principle cause for fractioning is because the effect of the radiotherapy is cumulative, so the treatment is fractioned to control the degree of tissue damage expressed in the 5Rs: reoxygenation, repair, redistribution, regeneration, and radiosensitivity [38]. Because in vitro studies deal with extracted and thus dead teeth, reoxygenation or repopulation is not possible. Therefore, it is possible to use a single dose of 60 Gy [28].

According to the literature (clinical research), composite resin restoration combined with fluoride therapy can prevent new caries lesions as efficiently as glass ionomer restoration [39]. For this reason, composite resin was the material of choice in this study.

The adhesive bonding potential can be impacted by variations in their chemical composition and the amounts of acidic monomers they contain [40]. Therefore, two different adhesives were evaluated in this study.

According to the results of the current study, the shear bond strength was affected harmfully by RT; G3 presented the poorest outcomes. Therefore, the first null hypothesis that ionizing radiation affects the dentin shear bond strength was rejected.

This could be attributed to the direct impact of 60 Gy of radiation on the structure of the dentin. It is known that bonding to dentin depends on the ability of the adhesive to infiltrate the exposed collagen fibrils and dentinal tubules formed by acid etching and the formation of a hybrid layer [41]. After ionizing radiation at different therapeutic doses, many investigations have revealed morphological alterations in enamel and dentin and changes in the organic and inorganic components of mineralized dental tissues [13]. When radiation and water interact, reactive oxygen species such as free radicals of hydrogen and hydrogen peroxide are created [42]. The production of free radicals and the process of radiolysis of water molecules can potentially cause cell death [43].

Since dentin has a high water content and ionizing radiation operates by creating free radicals when it reacts with water, these free radicals take electrons (they reduce themselves and oxidize the tissue) primarily from organic components [44] and may have adverse effects on the secondary and tertiary structures of dentin proteins. This could result in the loss of collagen fiber hydration and make the tissue drier and more friable [45].

This process can lead to certain micromorphological alterations in the dentin structure, such as fragmentation of collagen fibers [45] and dentinal tubule obliteration, which is followed by degeneration of odontoblast processes [46]. This can lead to failure in the formation of the hybrid layer and make the interface between the adhesive system and the irradiated dentin permeable [47].

Moreover, these reactive oxygen species have the potential to function as polymerization inhibitors for adhesive systems [17]. Therefore, the polymer matrix of the adhesive system might be more susceptible to hydrolytic destruction [48]. Therefore, this influences its primary bonding strength to dentin or enamel [17].

Furthermore, ionizing radiation influences the apatite crystals of the teeth, possibly interfering with adhesion [28]. Additionally, it can destroy the connection between the peritubular and intertubular dentine [13]. These changes in dentine biomechanical characteristics cause low bond strength since a porous adhesive interface is formed [13].

The results of the present study are in agreement with previous findings [8, 13, 14, 16, 17], which were carried out on permanent teeth, and revealed that when the adhesive restorations were carried out after radiotherapy, they had the lowest bond strength values.

The results of the present study contrast with those of D Mohenski et al. (2024) [49] and de Siqueira Mellara T, et al. (2020), (21) who reported that restorations placed before radiotherapy had the lowest shear bond strength. This may be linked to the use of different radiation sources, doses, and storage media.

However, the higher values in the preradiation group (G2) than in the group restored 24 h after RT (G3) indicated that the restoration-tooth bonding and the existing hybrid layer were unaffected by ionizing radiation.

These results are in agreement with those of other studies [16–18, 50] which revealed that the alterations in the dentin substrate caused by irradiation may not be significant enough to influence the performance of the preexisting hybrid layer and to impair the bonding interface.

On the other hand, in most of the studies, adhesive restoration was carried out immediately or 24 h after radiation exposure; however, clinically, patients are advised not to receive any elective dental treatment directly after exposure to radiotherapy. [51] Therefore, in our study, the restorative procedure was performed 6 months after radiation exposure to the teeth.

There was no significant difference among groups G1 and G4 (*p* > 0.001) regardless of the adhesive system applied. Because the samples were kept in distilled water for six months, some hydration likely occurred. As mentioned previously, RT has an adverse effect on the secondary and tertiary structures of dentin proteins, which results in the loss of collagen fiber hydration and makes the tissue drier and more friable. [45].

With respect to the adhesive system used, there was a relation between the adhesive system used and the shear bond strength to dentin.

The SB system, which is a self-etch adhesive system, presented superior values in this study, therefore the second null hypothesis that there is no difference between self-etch adhesive and etch and rinse adhesive was rejected.

This can be explained by the improved hydrolysis resistance and the chemical bonds generated by functional monomers [52]. Certain materials contain functional monomers, such as 10-MDP (10-methacryloyloxydecyl dihydrogen phosphate), which enable a more stable link to be formed with the calcium ions found in dental hydroxyapatite [53]. Furthermore, this type of adhesive avoids partial adhesive penetration in previously demineralized areas by utilizing hydrophilic acids to demineralize and permeate the dentin [54]. Additionally, the pH of the primers used with the self-etch adhesive is 2.0 [55]. As a result, the dentin partially demineralizes and a homogeneous hybrid layer with hydroxyapatite is formed, which can protect the collagen network [55–57].

Furthermore, dentin is completely demineralized by etch and rinse adhesive systems, which expose the collagen and leave the hydroxyapatite crystals vulnerable [19]. During dentin drying, collagen fibrils may collapse, which could prevent the resin monomers from penetrating the dentin sufficiently and weaken the bonding [58].

The results of the present study agreed with those of other studies [15, 19, 59], which investigated the bond strength of different adhesive systems after radiation and reported that self-etch adhesives provided greater bond strength to dentin than etch and rinse adhesives did.

However, Hanabusa et al. [59] investigated the shear bond strength of etch and rinse and self-etch adhesives and reported that there was no significant difference between these values irrespective of the radiation protocol.

The results of the current study contrast with those of MA Munoz et al. (2020) [22] and Sai et al. (2018) [21] who reported that the etch and rinse strategy has greater bond strength than the self-etch strategy. The cause of these discrepancies in the results may be due to differences in the stress distributions at the test interface between the two approaches [60], the difference in sample preparation methods and types of adhesive systems.

While the study successfully achieved its goals, it has several limitations. For example, it does not fully replicate in vivo conditions, such as oral hygiene and patient biology. The single-dose radiation exposure used in the study does not reflect the fractionated doses commonly used in clinical settings, which could potentially impact the study's results. Additionally, the study did not examine the long-term effects of radiotherapy beyond 6 months, so it is possible that more late effects could arise and progress over time. Therefore, we recommend conducting further in vivo studies with follow-up periods of more than 6 months to validate the findings.

One important implication of this study is that in clinical situations, the oral cavities of head and neck cancer (HNC) patients should be evaluated before radiotherapy begins. It is recommended to restore or extract their decayed teeth to prevent potential side effects of radiotherapy on dental tissues. Alternatively, teeth can be restored six months after the completion of radiotherapy.

## Conclusions

The following conclusions can be drawn from the results of this study:

1. The shear bond strength values of adhesive restorations performed 24 h after radiation were lower than those of the other groups.
2. The restoration performed after 6 months of radiation exposure had the same values of bond strength as teeth not exposed to radiation when the self-etch adhesive system was used in dentin.
3. Overall, 3M™ Single Bond Universal Adhesive, when used in “self-etch” mode presented better values in irradiated teeth than AdperSingle Bond 2 did.

## Supplementary Information

Supplementary Material 1.

Supplementary Material 2.

## Data Availability

The datasets used and analyzed during the current study are available from the corresponding author upon reasonable request.All data analyzed during this study are included in this published articles in the form of tables and figures.

## Abbreviations

RT: Radiotherapy
HNC: Head and neck cancer
PVC: Polyvinyl chloride
LED: Light emitting diode
Gy: Gray

## Glossary

Adhesion: State in which two surfaces are held together by chemical or physical forces or both with and without adhesive aid. Adhesion is one aspect of bonding.
Adhesive: Any substance that joins or creates close adherence of two or more surfaces. An intermediate material that causes two materials to adhere to each other.
Acid etching: Use of an acidic chemical substance to prepare the tooth enamel and or dentin surface to provide retention for bonding.
Primary teeth: The first teeth to erupt in a pediatric patient.
Bolus: A rubber material which has properties equivalent to those of human tissue when irradiated.
Cancer: A general term for more than 100 diseases that have uncontrolled, abnormal growth of cells that can invade and destroy healthy tissues.
Cobalt 60: A radioactive substance sometimes used as a radiation source to treat cancer.
Chisel: A metal tool with a cutting edge at the end of a blade.
Dentin: Hard tissue which forms the bulk of the tooth and develops from the dental papilla and dental pulp and is in the mature state is mineralized.
Etch-and-rinse adhesives: These adhesives utilize the use of acid etching of both enamel and dentin and the application of both primer and adhesive in separate steps or the application of the primer/adhesive mixture in a single bonding step.
Fractionation: Dividing the total dose of radiation into smaller doses to give healthy tissue time to repair itself.
Gamma Rays: High-energy rays that come from a radioactive source such as cobalt-60.
Gray: A measurement of the amount of radiation dose absorbed by the body (1 Gray = 100 rads).
Radiation Therapy: The use of high-energy penetrating rays or subatomic particles to treat disease. Types of radiation include x-ray, conformal, electron beam, alpha and beta particle, and gamma ray. Radioactive substances include cobalt, radium, iridium cesium, iodine, and palladium.
Self-etch adhesives: Adhesive that contain acidic monomers, which etch and prime the tooth simultaneously.
Scaler: An instrument used to remove plaque and calculus from the surfaces of teeth during professional cleanings.
Phantom: A device or object specifically designed to mimic human anatomy or tissue properties.
Polyvinyl Chloride (PVC): Is a high-strength thermoplastic material. It is widely used in applications such as pipes, medical devices, and wire.
Scattering radiation: Radiation that spreads out in different directions from a radiation beam when the beam interacts with a substance, such as body tissue.
Tygon Tube: Is a flexible plastic polymer tube with various formulations.
X-ray: One form of radiation that can be used at low levels to produce an image of the body on film or at high levels to destroy cancer cells.

## References

[1] Siegel RL, Giaquinto AN, Jemal A. Cancer statistics, 2024. CA: a cancer journal for clinicians. 2024;74(1):12–49. 10.3322/caac.21820. 10.3322/caac.21820 38230766

[2] Gormley M, Creaney G, Schache A, Ingarfield K, Conway DI. Reviewing the epidemiology of head and neck cancer: definitions, trends and risk factors. Br Dent J. 2022;233(9):780–6. 10.1038/s41415-022-5166-x. 36369568 10.1038/s41415-022-5166-x PMC9652141

[3] Schmidt Jensen J, Grønhøj C, Ruud Kjær EK, Charabi BW, von Buchwald C, Hjuler T. Second primary cancers in pediatric head and neck cancer survivors in Denmark during 1980–2014: A nationwide study. Int J Pediatr Otorhinolaryngol. 2019Dec;127: 109648. 10.1016/j.ijporl.2019.109648. 31472358 10.1016/j.ijporl.2019.109648

[4] Carini F, Bucalo C, Saggese V, Monai D, Porcaro G. Case control study to assess the possibility of decrease the risk of osteoradionecrosis in relation to the dose of radiation absorbed by the jaw. Annali Di Stomatologia. 2012;3(2 Suppl):3–7 pmid: 23285316. 23285316 PMC3512551

[5] Ma SJ, Rivers CI, Serra LM, Singh AK. Long-term outcomes of interventions for radiation-induced xerostomia: A review. World journal of clinical oncology. 2019;10(1):1–13. 10.5306/wjco.v10.i1.1. 30627521 10.5306/wjco.v10.i1.1 PMC6318483

[6] Joshi VK. Dental treatment planning and management for the mouth cancer patient. Oral Oncol. 2010;46(6):475–9. 10.1016/j.oraloncology.2010.03.010. 20400359 10.1016/j.oraloncology.2010.03.010

[7] De Moor RJG, Stassen IG, van ’t Veldt Y, Torbeyns D, Hommez GMG. Two-year clinical performance of glass ionomer and resin composite restorations in xerostomic head- and neck-irradiated cancer patients. Clin Oral Investig. 2011;15(1):31–8. 10.1007/s00784-009-0355-4. 19997859 10.1007/s00784-009-0355-4

[8] Madrid Troconis CC, Santos-Silva AR, Brandão TB, Lopes MA, de Goes MF. Impact of head and neck radiotherapy on the mechanical behavior of composite resins and adhesive systems: A systematic review. Dental materials : official publication of the Academy of Dental Materials. 2017;33(11):1229–43. 10.1016/j.dental.2017.07.014. 28801178 10.1016/j.dental.2017.07.014

[9] Gwinnett AJ, Dickerson WG, Yu S. Dentin bond shear strength and microleakage for Syntac/Heliomolar: a comparison between the manufacturer’s and total etch technique. J Esthet Dent. 1992;4(5):164–8. 10.1111/j.1708-8240.1992.tb00688.x. 1288602 10.1111/j.1708-8240.1992.tb00688.x

[10] Akasapu A, Hegde U, Murthy PS. Enamel Surface Morphology: An Ultrastructural Comparative Study of Anterior and Posterior Permanent Teeth. J Microsc Ultrastruct. 2018;6(3):160–4 PMID: 30221142. 30221142 10.4103/JMAU.JMAU_27_18 PMC6130241

[11] De Menezes Oliveira MAH, Torres CP, Gomes-Silva JM, Chinelatti MA, De Menezes FCH, Palma-Dibb RG, et al. Microstructure and mineral composition of dental enamel of permanent and deciduous teeth. Microsc Res Tech. 2010May;73(5):572–7. 10.1002/jemt.20796. 19937744 10.1002/jemt.20796

[12] Lakomaa EL, Rytömaa I. Mineral composition of enamel and dentin of primary and permanent teeth in Finland. Scand J Dent Res. 1977;85(2):89–95. 10.1111/j.1600-0722.1977.tb00537.x. 265089 10.1111/j.1600-0722.1977.tb00537.x

[13] Rodrigues RB, Soares CJ, Junior PCS, Lara VC, Arana-Chavez VE, Novais VR. Influence of radiotherapy on the dentin properties and bond strength. Clin Oral Invest. 2018;22(2):875–83. 10.1007/s00784-017-2165-4. 10.1007/s00784-017-2165-4 28776096

[14] Freitas Soares E, Zago Naves L, Bertolazzo Correr A, Costa AR, Consani S, Soares CJ, et al. Effect of radiotherapy, adhesive systems and doxycycline on the bond strength of the dentin-composite interface. Am J Dent. 2016Dec;29(6):352–6 PMID: 29178724. 29178724

[15] de Cunha SRB, Ramos PAMM, Haddad CMK, da Silva JLF, Fregnani ER, Aranha ACC. Effects of Different Radiation Doses on the Bond Strengths of Two Different Adhesive Systems to Enamel and Dentin. J Adhes Dent. 2016;18(2):151–6. 27022644 10.3290/j.jad.a35841

[16] Naves LZ, Novais VR, Armstrong SR, Correr-Sobrinho L, Soares CJ. Effect of gamma radiation on bonding to human enamel and dentin. Supportive care in cancer : official journal of the Multinational Association of Supportive Care in Cancer. 2012;20(11):2873–8. 10.1007/s00520-012-1414-y. 22415607 10.1007/s00520-012-1414-y

[17] Yadav S, Yadav H. Ionizing irradiation affects the microtensile resin dentin bond strength under simulated clinical conditions. Journal of conservative dentistry : JCD. 2013;16(2):148–51 PMID: 23716968. 23716968 10.4103/0972-0707.108198 PMC3659861

[18] Gernhardt CR, Kielbassa AM, Hahn P, Schaller HG. Tensile bond strengths of four different dentin adhesives on irradiated and non-irradiated human dentin in vitro. J Oral Rehabil. 2001;28(9):814–20. 10.1111/j.1365-2842.2001.00758.x. 11580819 10.1046/j.1365-2842.2001.00758.x

[19] Arid J, Palma-Dibb RG, de Oliveira HF, Nelson-Filho P, de Carvalho FK, da Silva LAB, et al. Radiotherapy impairs adhesive bonding in permanent teeth. Supportive care in cancer : official journal of the Multinational Association of Supportive Care in Cancer. 2020;28(1):239–47. 10.1007/s00520-019-04782-5. 31020438 10.1007/s00520-019-04782-5

[20] Mellara T de S, Paula-Silva FWG, Arid J, de Oliveira HF, Nelson-Filho P, Bezerra Silva RA, et al. Radiotherapy Impairs Adhesive Bonding in Primary Teeth: An In Vitro Study. Journal of dentistry for children (Chicago, Ill). 2020;87(2):69–76. PMID: 32787999. 32787999

[21] Sai K, Takamizawa T, Imai A, Tsujimoto A, Ishii R, Barkmeier WW, et al. Influence of Application Time and Etching Mode of Universal Adhesives on Enamel Adhesion. Journal of Adhesive Dentistry. 2018;20(1). PMID: 29507919. 10.3290/j.jad.a39913 29507919

[22] Muñoz MA, Garín-Correa C, González-Arriagada W, Quintela Davila X, Häberle P, Bedran-Russo A, et al. The adverse effects of radiotherapy on the structure of dental hard tissues and longevity of dental restoration. Int J Radiat Biol. 2020;96(7):910–8. 10.1080/09553002.2020.1741718. 32159405 10.1080/09553002.2020.1741718

[23] Oglakci B, Burduroğlu D, Eriş AH, Mayadağlı A, Arhun N. How Does Radiotherapy Affect the Adhesion of Universal Adhesive to Enamel and Dentin? A Qualitative and Quantitative Analysis? Odovtos - International Journal of Dental Sciences. 2022 1;24(3 SE-Original Basic Research Articles):75–90. 10.15517/ijds.2022.50665.

[24] Cohen J. Statistical Power Analysis for the Behavioral Sciences (2nd ed.). 1988. 567 p. 10.4324/9780203771587.

[25] Moore C, Killough S, Markey N, McLister C, McKenna G. Impact of Referral Protocols on the Dental Management of Patients Undergoing Treatment for Head and Neck Oncology in Northern Ireland. Eur J Prosthodont Restor Dent. 2016;24(1):19–22 PMID: 27039474. 27039474

[26] Team RC. R Core Team 2023 R: A language and environment for statistical computing. R foundation for statistical computing. R Foundation for Statistical Computing. 2023. Available from: https://www.r-project.org/.

[27] Geneva. ISO.Guidance on testing of adhesion to tooth structure. International Organization for Standardization. Switzerland; 1994. p. 1–4.

[28] Biscaro SL, Moraes RR, Correr AB, Almeida SM, Bóscolo FN, Soares CJ, et al. Effect of X-ray radiation dose on the bond strength of different adhesive systems to dentin. J Adhes Dent. 2009;11(5):355–60 PMID: 19841761. 19841761

[29] Dibo da Cruz A, Goncalves L de S, Rastelli AN de S, Correr-Sobrinho L, Bagnato VS, Boscolo FN. Bond strength of dental adhesive systems irradiated with ionizing radiation. J Adhes Dent. 2010;12(2):123–8. PMID: 20157673. 10.3290/j.jad.a17530 20157673

[30] Albright JT, Topham AK, Reilly JS. Pediatric head and neck malignancies: US incidence and trends over 2 decades. Archives of Otolaryngology--Head & Neck Surgery. 2002;128(6):655–9. PMID: 12049559. 10.1001/archotol.128.6.655 12049559

[31] Al-Nawas B, Al-Nawas K, Kunkel M, Grötz KA. Quantifying radioxerostomia: salivary flow rate, examiner’s score, and quality of life questionnaire. Strahlentherapie und Onkologie : Organ der Deutschen Rontgengesellschaft. 2006;182(6):336–41. 10.1007/s00066-006-1508-x. 10.1007/s00066-006-1508-x 16703289

[32] Vissink A, Burlage FR, Spijkervet FKL, Jansma J, Coppes RP. Prevention and treatment of the consequences of head and neck radiotherapy. Crit Rev Oral Biol Med. 2003;14(3):213–25. 10.1177/154411130301400306. 12799324 10.1177/154411130301400306

[33] Kumar N, Brooke A, Burke M, John R, O’Donnell A, Soldani F. The oral management of oncology patients requiring radiotherapy, chemotherapy and/or bone marrow transplantation. Faculty Dent J. 2013;4(4):200–3. 10.1308/204268513X13776914744952.

[34] Lieshout HFJ, Bots CP. The effect of radiotherapy on dental hard tissue–a systematic review. Clin Oral Invest. 2014;18(1):17–24. 10.1007/s00784-013-1034-z. 10.1007/s00784-013-1034-z 23873320

[35] Mallick I, Waldron JN. Radiation therapy for head and neck cancers. Seminars Oncol Nurs J. 2009;25(3):193–202. 10.1016/j.soncn.2009.05.002. 10.1016/j.soncn.2009.05.002 19635398

[36] Marur S, Forastiere AA. Head and neck cancer: changing epidemiology, diagnosis, and treatment. Mayo Clin Proc. 2008;83(4):489–501. 10.4065/83.4.489. 18380996 10.4065/83.4.489

[37] Harrington K, Jankowska P, Hingorani M. Molecular biology for the radiation oncologist the 5Rs of radiobiology meet the hallmarks of cancer. Clin Oncol (R Coll Radiol (Great Britain)). 2007;19(8):561–71. 10.1016/j.clon.2007.04.009. 10.1016/j.clon.2007.04.009 17591437

[38] Widikusumo A, Purnamawati S. Six fractions weekly as accelerated fraction radiotherapy: Is it applicable for nasopharyngeal cancer? A review Contemporary oncology. 2019;23(3):127–32. 10.5114/wo.2019.89240. 31798326 10.5114/wo.2019.89240 PMC6883960

[39] McComb D, Erickson RL, Maxymiw WG, Wood RE. A clinical comparison of glass ionomer, resin-modified glass ionomer and resin composite restorations in the treatment of cervical caries in xerostomic head and neck radiation patients. Oper Dent. 2002;27(5):430–7 PMID: 12216559. 12216559

[40] da Rosa WL, Piva E, da Silva AF. Bond strength of universal adhesives: A systematic review and meta-analysis. J Dent. 2015;43(7):765–76. 10.1016/j.jdent.2015.04.003. 25882585 10.1016/j.jdent.2015.04.003

[41] Pashley DH, Tay FR, Breschi L, Tjäderhane L, Carvalho RM, Carrilho M, et al. State of the art etch-and-rinse adhesives. Dental materials : official publication of the Academy of Dental Materials. 2011;27(1):1–16. 10.1016/j.dental.2010.10.016. 21112620 10.1016/j.dental.2010.10.016 PMC3857593

[42] Cole T, Silver AH. Production of Hydrogen atoms in teeth by X-Irradiation. Nature. 1963;200:700–1. 10.1038/200700a0. 14109974 10.1038/200700a0

[43] Ogawa Y. Paradigm Shift in Radiation Biology/Radiation Oncology-Exploitation of the “H_2_O_2_ Effect” for Radiotherapy Using Low-LET (Linear Energy Transfer) Radiation such as X-rays and High-Energy Electrons. Cancers. 2016;8(3). 10.3390/cancers8030028. 10.3390/cancers8030028 PMC4810112 26927177

[44] Pioch T, Golfels D, Staehle HJ. An experimental study of the stability of irradiated teeth in the region of the dentinoenamel junction. Endod Dent Traumatol. 1992;8(6):241–4. 10.1111/j.1600-9657.1992.tb00251.x. 1302687 10.1111/j.1600-9657.1992.tb00251.x

[45] Gonçalves LMN, Palma-Dibb RG, Paula-Silva FWG, de Oliveira HF, Nelson-Filho P, da Silva LAB, et al. Radiation therapy alters microhardness and microstructure of enamel and dentin of permanent human teeth. J Dent. 2014;42(8):986–92. 10.1016/j.jdent.2014.05.011. 24887361 10.1016/j.jdent.2014.05.011

[46] Fisher BV, Morgan RE, Phillips GO, Wardale HW. Radiation damage in calcium phosphates and collagen: an interpretation of ESR spectra. Radiat Res. 1971May;46(2):229–35 PMID: 4327623. 4327623

[47] Grötz KA, Duschner H, Kutzner J, Thelen M, Wagner W. [New evidence for the etiology of so-called radiation caries. Proof for directed radiogenic damage od the enamel-dentin junction]. Strahlentherapie und Onkologie : Organ der Deutschen Rontgengesellschaft . 1997;173(12):668–76. 10.1007/BF03038449. 10.1007/BF03038449 9454351

[48] Rónai E, Benkö G. Effect of acute 60Co-gamma-irradiation on the in vivo lipid peroxidation in experimental animals. Acta Physiol Hung. 1984;63(1):13–9 PMID: 6741554. 6741554

[49] Mohenski D, Vrebac M, Sever EK, Grego T, Goršeta K, Ivanišević A. Effects of Ionizing Radiation on the Shear Bond Strength of Composite Materials to Dentin. Vol. 8, Journal of Composites Science. 2024. 10.3390/jcs8070261.

[50] Galetti R, Santos-Silva AR, da Antunes ANG, de Alves FA, Lopes MA, de Goes MF. Radiotherapy does not impair dentin adhesive properties in head and neck cancer patients. Clin Oral Investi. 2014;18(7):1771–8. 10.1007/s00784-013-1155-4. 10.1007/s00784-013-1155-4 24309632

[51] Devi S, Singh N. Dental care during and after radiotherapy in head and neck cancer. National journal of maxillofacial surgery. 2014;5(2):117–25 PMID: 25937720. 25937720 10.4103/0975-5950.154812 PMC4405951

[52] Bernard C, Villat C, Abouelleil H, Gustin MP, Grosgogeat B. Tensile Bond Strengths of Two Adhesives on Irradiated and Nonirradiated Human Dentin. Biomed Res Int. 2015;2015: 798972. 10.1155/2015/798972. 26783528 10.1155/2015/798972 PMC4689887

[53] Van Meerbeek B, Yoshihara K, Yoshida Y, Mine A, De Munck J, Van Landuyt KL. State of the art of self-etch adhesives. Dental materials : official publication of the Academy of Dental Materials. 2011;27(1):17–28. 10.1016/j.dental.2010.10.023. 21109301 10.1016/j.dental.2010.10.023

[54] Bulucu B, Avsar A, Demiryürek EO, Yesilyurt C. Effect of radiotherapy on the microleakage of adhesive systems. J Adhes Dent. 2009;11(4):305–9 PMID: 19701512. 19701512

[55] Peumans M, De Munck J, Van Landuyt KL, Poitevin A, Lambrechts P, Van Meerbeek B. Eight-year clinical evaluation of a 2-step self-etch adhesive with and without selective enamel etching. Dental materials : official publication of the Academy of Dental Materials. 2010;26(12):1176–84. 10.1016/j.dental.2010.08.190. 20947155 10.1016/j.dental.2010.08.190

[56] De Munck J, Van Landuyt K, Peumans M, Poitevin A, Lambrechts P, Braem M, et al. A critical review of the durability of adhesion to tooth tissue: methods and results. J Dent Res. 2005;84(2):118–32. 10.1177/154405910508400204. 15668328 10.1177/154405910508400204

[57] Van Landuyt KL, Kanumilli P, De Munck J, Peumans M, Lambrechts P, Van Meerbeek B. Bond strength of a mild self-etch adhesive with and without prior acid-etching. J Dent. 2006;34(1):77–85. 10.1016/j.jdent.2005.04.001. 15979226 10.1016/j.jdent.2005.04.001

[58] Leite ML, Charamba C, Lima R, Meireles S, Duarte R, Andrade A. Bond strength of universal adhesive applied to dry and wet dentin. Brazilian J Oral Sci. 2020;9(19):e201662. 10.20396/bjos.v19i0.8658220.

[59] Hanabusa M, Kimura S, Hori A, Yamamoto T. Effect of irradiation source on the dentin bond strength of a one-bottle universal adhesive containing an amide monomer. J Adhes Sci Technol. 2019;33(20):2265–80. 10.1080/01694243.2019.1639588.

[60] Scherrer SS, Cesar PF, Swain MV. Direct comparison of the bond strength results of the different test methods: a critical literature review. Dental materials : official publication of the Academy of Dental Materials. 2010;26(2):e78-93. 10.1016/j.dental.2009.12.002. 20060160 10.1016/j.dental.2009.12.002

