# Supplementary figures and images for: Effect of ionizing radiation on the shear bond strength of two different adhesive systems in primary teeth. in-vitro study
Source: BMC Oral Health. 2024 Oct 21;24:1261. doi: 10.1186/s12903-024-04996-y (PMC11494816; doi:10.1186/s12903-024-04996-y)

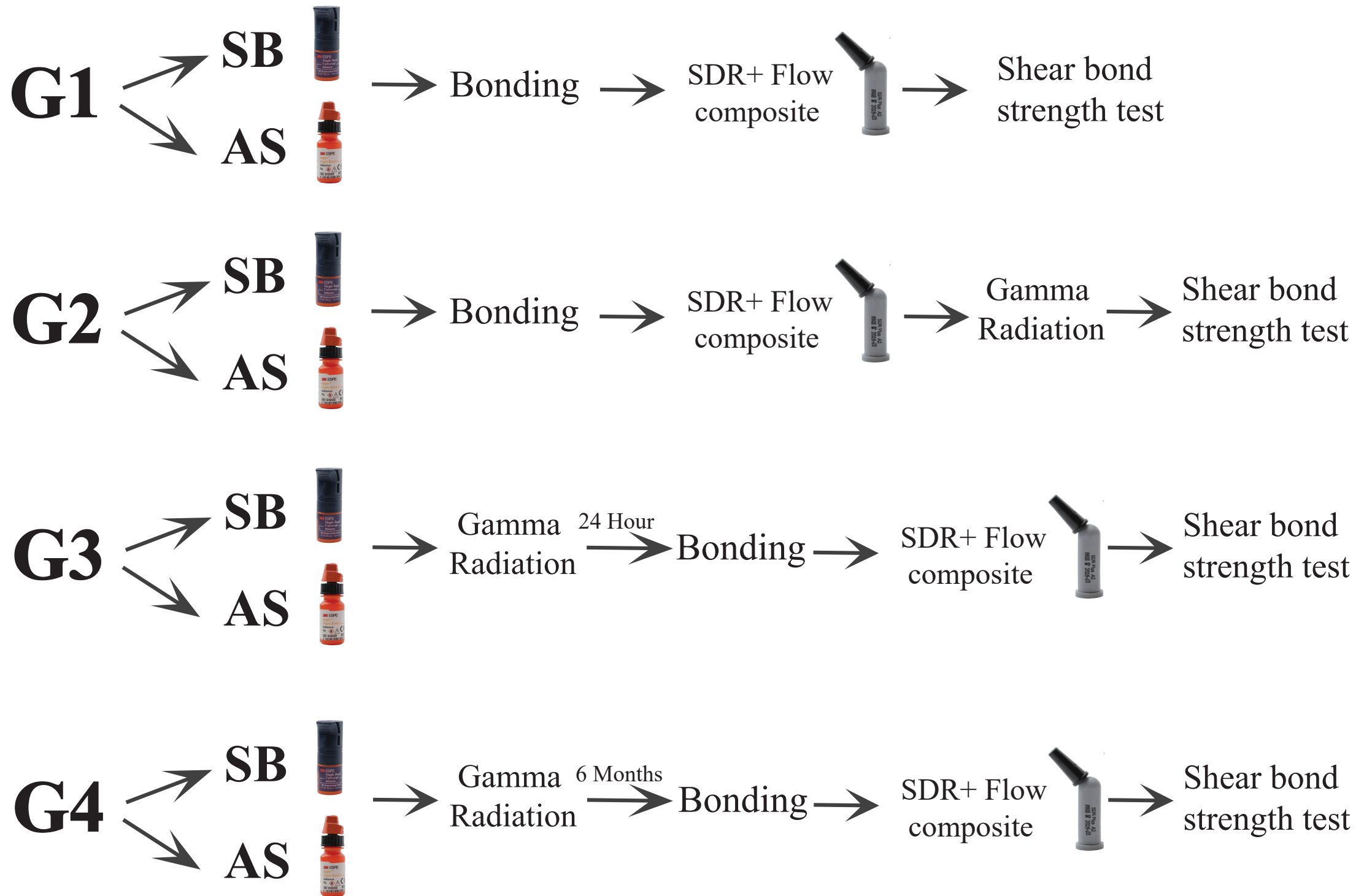

Supplement: Supplementary file 1 — Supplementary Material 1. [file 12903_2024_4996_MOESM1_ESM.pdf]
