# Supplementary material for: Effect of ionizing radiation on the shear bond strength of two different adhesive systems in primary teeth. in-vitro study
Source: BMC Oral Health. 2024 Oct 21;24:1261. doi: 10.1186/s12903-024-04996-y (PMC11494816; doi:10.1186/s12903-024-04996-y)
Supplement: Supplementary file 2 — Supplementary Material 2. [file 12903_2024_4996_MOESM2_ESM.pdf]

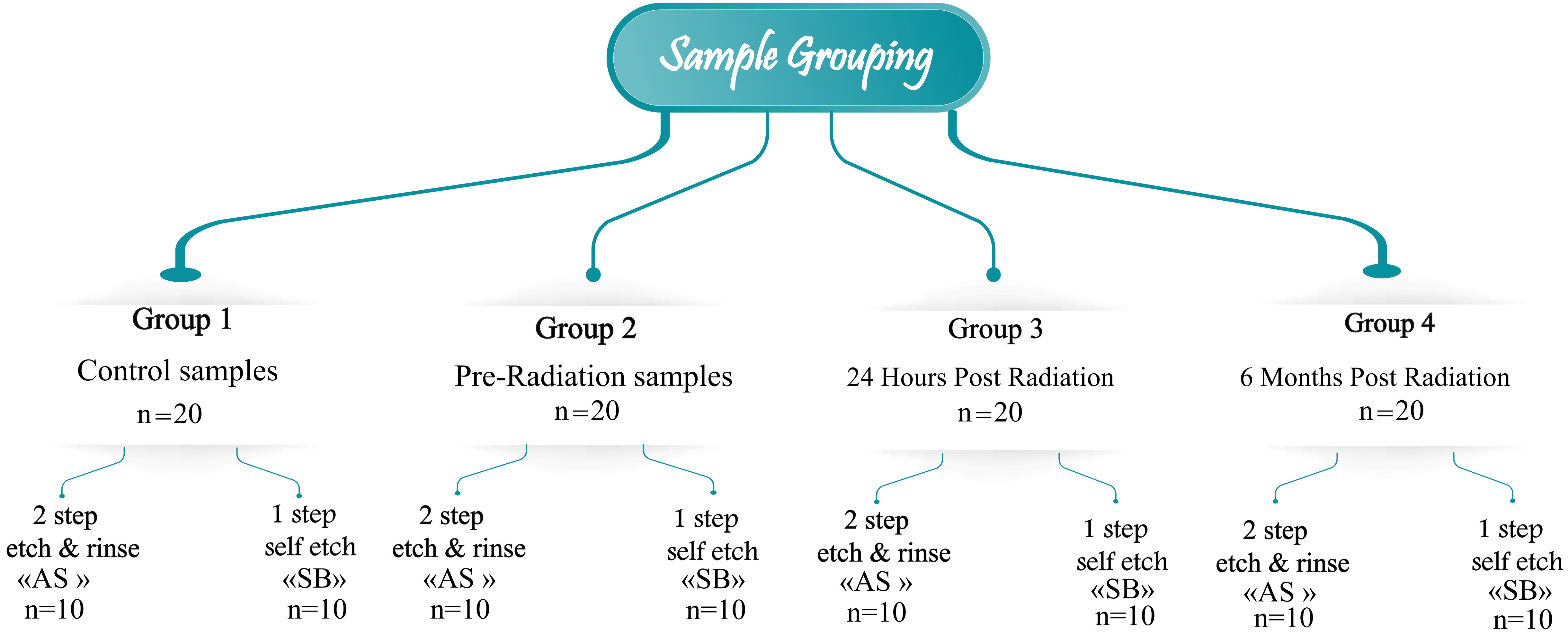
Sample Grouping
Group 1
Control samples
n=20
Group 2
Pre-Radiation samples
n=20
Group 3
24 Hours Post Radiation
n=20
Group 4
6 Months Post Radiation
n=20
2 step
etch & rinse
«AS »
n=10
1 step
self etch
«SB»
n=10
2 step
etch & rinse
«AS »
n=10
1 step
self etch
«SB»
n=10
2 step
etch & rinse
«AS »
n=10
1 step
self etch
«SB»
n=10
2 step
etch & rinse
«AS »
n=10
1 step
self etch
«SB»
n=10
